# *In vitro *antineoplastic activity in triple-negative breast cancer cell line and *in vivo*

**DOI:** 10.1186/1753-6561-8-S4-P22

**Published:** 2014-10-01

**Authors:** Klesia Madeira, Murilo Cerri, Renata Daltoé, Alice Herlinger, João Allochio Filho, Sandro Greco, Leticia Rangel

**Affiliations:** 1 Biotechnology Program/RENORBIO, Federal University of Espirito Santo (UFES), ES, Brazil; 2 Department of Chemistry, Federal University of Espirito Santo (UFES), ES, Brazil; 3 Department of Pharmaceutical Sciences, Federal University of Espirito Santo (UFES), ES, Brazil

## Background

Triple negative breast cancer (TNBC) is a heterogeneous subgroup (ER-, PR-, and HER2-) of invasive breast cancer, associated to poor prognosis, partially due to its resistance to available drugs. Therefore, it is imperative to discover new treatment options for the disease. In this context, we have synthesized and screened novel naphtoquinone-derived drugs (patent-protected), rationally designed to act through multiple pathways to avoid tumor chemoresistance.

## Methods

Drugs antineoplastic efficacy (AE) was accessed in the claudin-low TNBC cell line, MDA-MB231, by cellular metabolic viability (CMV) and IC_50 _calculation (MTT method; GraphPad Prism version 5.1). Drugs toxicity was studied in healthy mice, following the Guideline 423 (for test of chemicals) of OECD; blood cells and tissues were analyzed by a Pathologist. Computational molecular dock studies were conducted to investigate the molecules tridimensional conformation and bounding energy to topoisomerase 2 (TOPO) and PI3K (Autodock Vina software).

## Results and conclusions

We screened the AE of 43 novel drugs in MDA-MB231 (CMV≤50% with 7 drugs). Of these, the most promising drugs PIC 20 (IC_50 _1.38x10^-5^M; CMV = 10%) and PIC21 (IC_50 _5.00x10^-5^M; CMV = 30%) showed significantly higher AE than cisplatin (IC_50 _1.56x10^-4^M; CMV>90%), doxorubicin (IC_50 _1.76x10^-4^M; CMV = 62%), and paclitaxel (IC_50 _5.05x10^-7^M; CMV = 80%). None of the treated mice died, neither demonstrated symptoms of toxicity, following 14-days treatment with PIC. Indeed, there was no significant change in the animals' weight and general activity/behavior. Major organs showed no significant morphological changes, congestion, edema, necrosis, degeneration or inflammation. On the other hand, there was a 48.98% decrease in their hematocrit count. Finally, based on the crystalline structure of proteins deposited on PDB (1QZR, TOPO; 1E7U, PI3K), and PIC20 and PIC21 tridimensional structures, we concluded that the novel molecules bind to the ATP domain of the proteins with similar interaction energy (E) than the TOPO - Doxorubicin (E = -5.6) and Etoposide (E = -5.7) - or PI3K inhibitors - LY294002 (E = -9.5) and Wortmannin (E = -8.8): PIC20: E = -5.3 and -8.9; PIC21: E = -5.7 and -8.2, for TOPO and PI3K, respectively. In conclusion, we present novel and potentially safe drugs to treat TNBC, in an innovative and economically viable approach.

